# Contributors to the *RSC Chemical Biology* Emerging Investigators Collection 2022

**DOI:** 10.1039/d2cb90028a

**Published:** 2022-08-01

## Abstract

This article profiles the early career researchers whose work features in the *RSC Chemical Biology* Emerging Investigators Collection 2022.
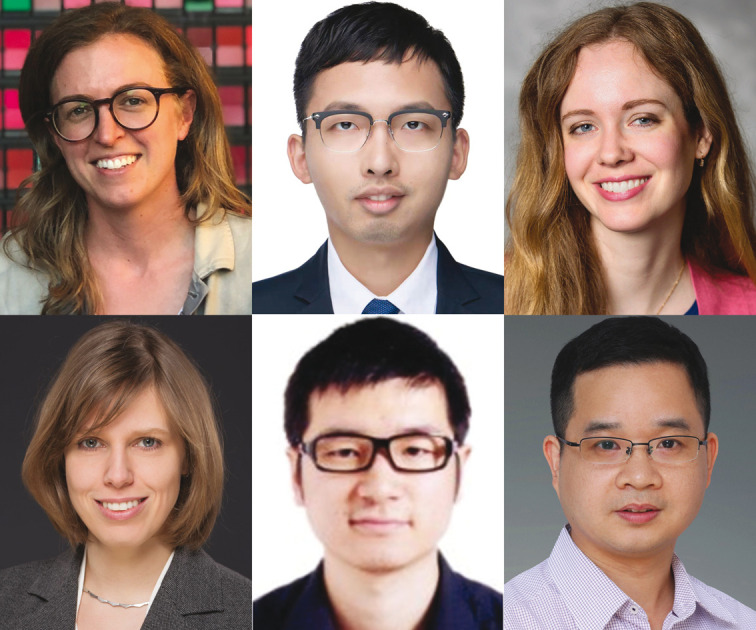


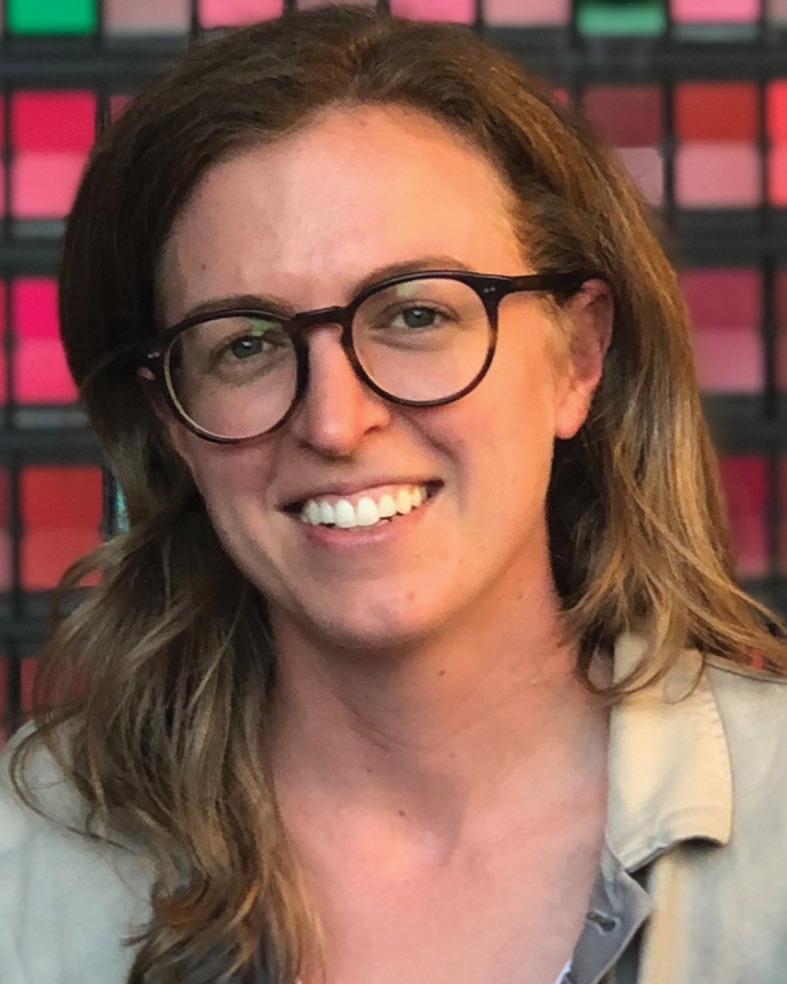
Balyn Zaro, PhD, is an Assistant Professor at UC San Francisco. Her lab leverages chemical biology and proteomics to study the innate immune system. She is particularly interested in how macrophages recognize self and non-self. She completed her PhD studies in chemical biology in the laboratory of Matthew Pratt, PhD, where she developed chemical reporters of protein glycosylation. For her postdoctoral studies, she worked in the laboratory of Ben Cravatt, PhD, utilizing chemical proteomics to characterize protein reactivity of covalent drugs. Prior to the start of her independent career, she gained additional training in innate immunity with Irving Weissman, MD.

Her contribution to the 2022 *RSC Chemical Biology Emerging Investigators* collection can be read at https://doi.org/10.1039/D2CB00076H


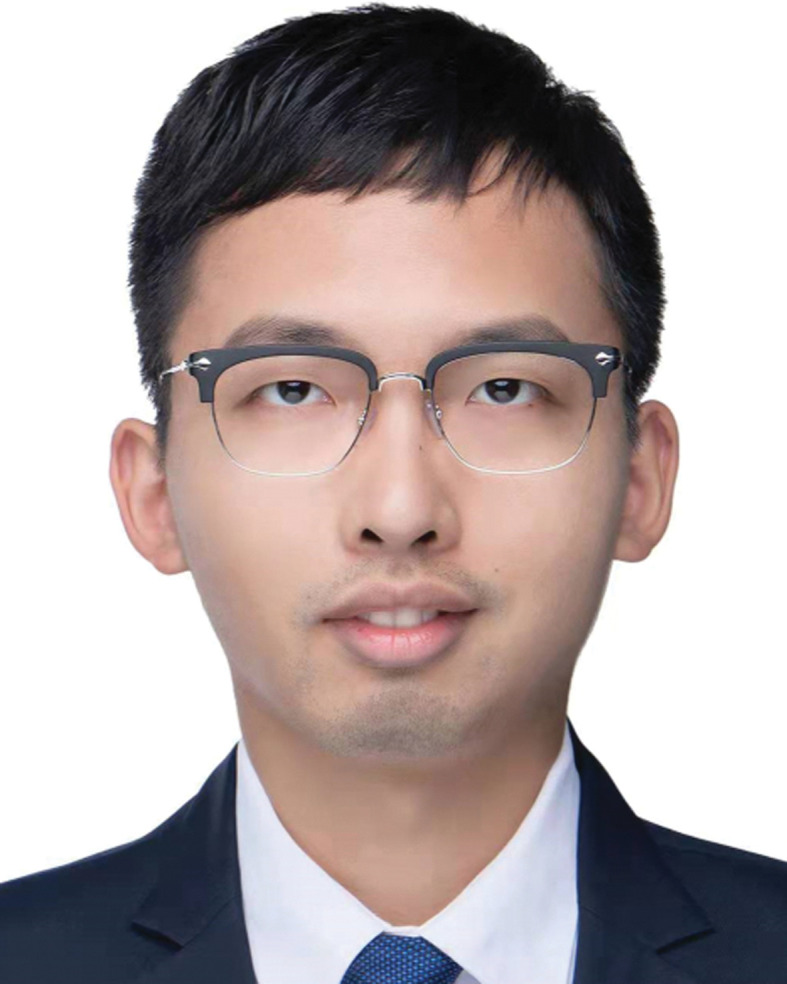
Dr Jie P. Li is a Group Leader at the Chemistry and Biomedicine Innovation Center (ChemBIC), School of Chemistry and Chemical Engineering, Nanjing University, China. After completing his DPhil degree in 2015 at Peking University, China, he undertook postdoctoral work at the Scripps Research Institute, USA. He started his independent research career in 2018 and his research group interest is focused on the topic of “chemical single-cell omics”, which utilizes chemical tools to study intracellular and intercellular signaling at the single-cell level for understanding the immune system.

His contribution to the 2022 *RSC Chemical Biology Emerging Investigators* collection can be read at https://doi.org/10.1039/D2CB00046F


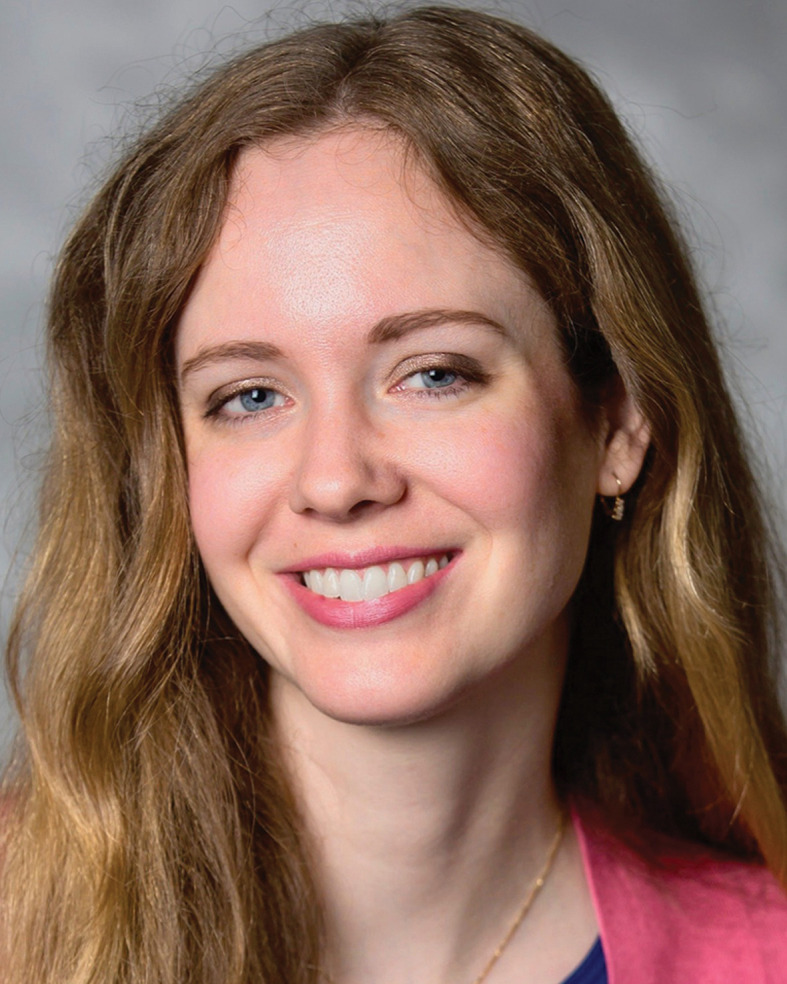
Katherine M. Davis is an Assistant Professor of Chemistry at Emory University. She obtained her BS in physics and mathematics from the University of Louisville, followed by a PhD in physics from Purdue University under the guidance of Prof. Yulia Pushkar. After a short postdoc in the Ando lab at Princeton University, she conducted further postdoctoral training jointly mentored by Prof. John T. Groves at Princeton and Prof. Amie Boal at the Pennsylvania State University. Her lab is broadly interested in developing and applying time-resolved physical methods for decoding the structure and function of metalloenzymes.

Her contribution to the 2022 *RSC Chemical Biology Emerging Investigators* collection can be read at https://doi.org/10.1039/D1CB00255D


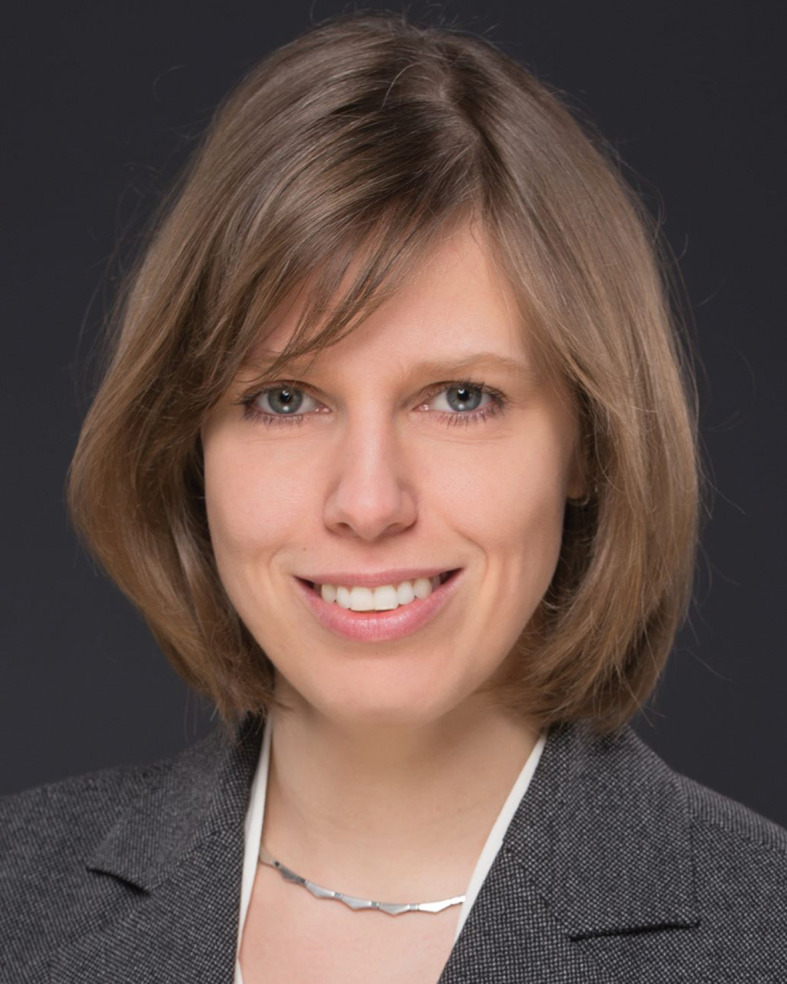
Franziska Thomas studied chemistry at Humboldt University Berlin, where she received her PhD in 2010. After a postdoctoral stay at the University of Bristol, UK, where she worked with Dek Woolfson, she became an independent research group leader at the University of Göttingen in 2015. In 2019, she became a junior professor of organic chemistry at the University of Heidelberg. Her research interests include protein design, *de novo* design of functional peptide entities, and identification of small molecules and peptides as drug candidates for the potential treatment of amyotrophic lateral sclerosis.

Her contribution to the 2022 *RSC Chemical Biology Emerging Investigators* collection can be read at https://doi.org/10.1039/D1CB00252J


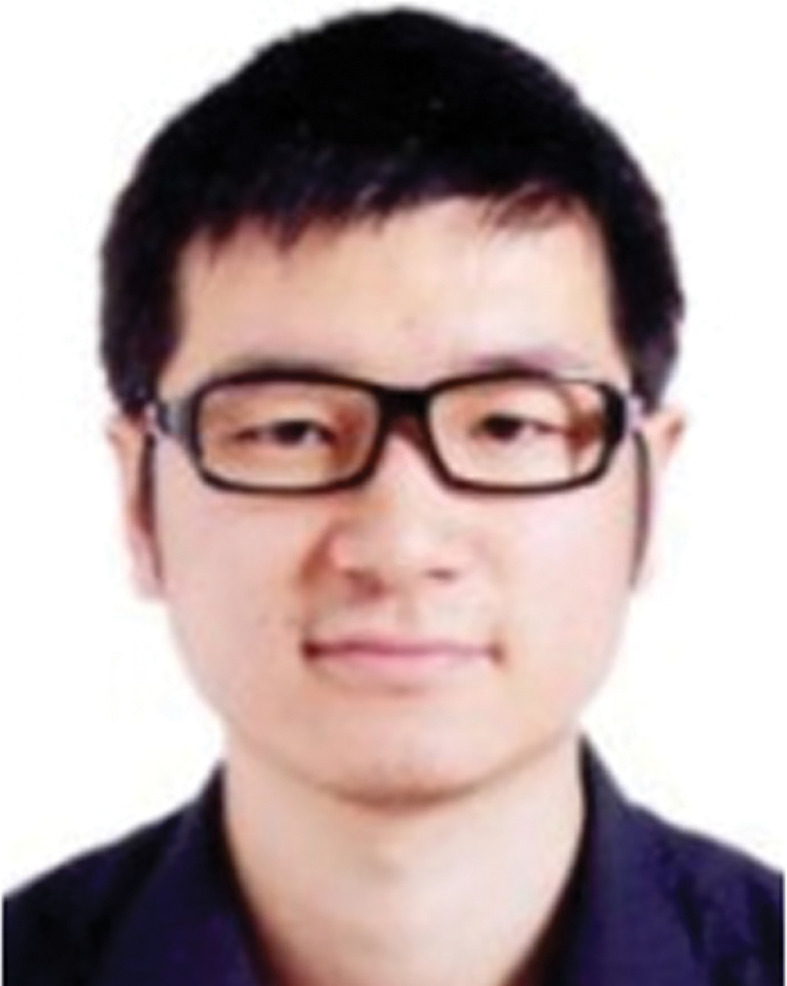
Dr Ran Xie completed his undergraduate degree in polymer chemistry from Shanghai Jiaotong University in 2010. He then obtained his PhD in Chemical Glycobiology from Peking University under the supervision of Prof. Xing Chen in 2015. After completing postdoctoral work at Harvard University in collaboration with Prof. Daniel Kahne in the field of Microbiology and Glycobiology, he joined Nanjing University in December 2019 as a principal investigator in the School of Chemistry and Chemical Engineering (SCCE) and Chemistry and Biomedicine Innovation Center (ChemBIC). Dr Xie's current research interest is focused on developing novel chemical methods for functional study of glycans.

His contribution to the 2022 *RSC Chemical Biology Emerging Investigators* collection can be read at https://doi.org/10.1039/D2CB00072E


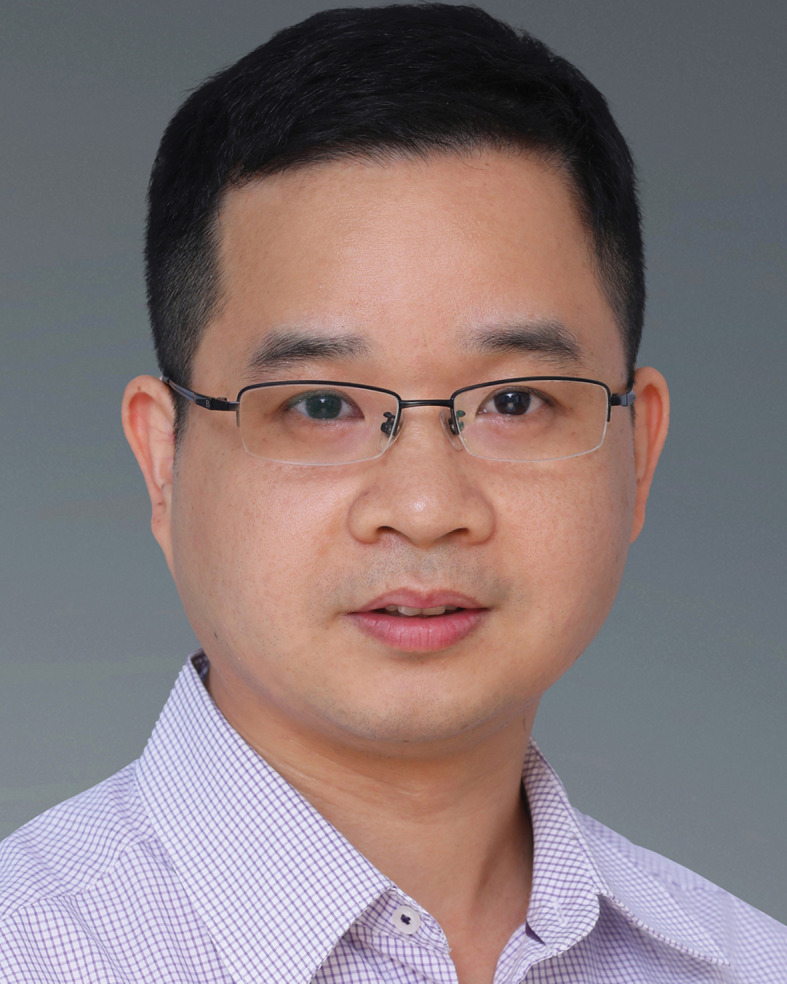
Jinhao Gao obtained his BSc from Nanjing University in 2004 and his PhD from The Hong Kong University of Science and Technology (HKUST) in 2008. He was a postdoctoral fellow at Stanford University from 2008 to 2010. He is now a professor of chemical biology at Xiamen University. He was awarded the National Natural Science Fund for Excellent Young Scholars (2012) and Distinguished Young Scholars (2021). His main research interests include chemical biology, nanochemistry, MRI probes, molecular imaging, and cancer therapy.

His contribution to the 2022 *RSC Chemical Biology Emerging Investigators* collection can be read at https://doi.org/10.1039/D1CB00257K

## Supplementary Material

